# Frustration: A Challenge in Chronic Conditions

**DOI:** 10.5812/nms.14166

**Published:** 2013-09-15

**Authors:** Negin Masoudi Alavi

**Affiliations:** 1 Trauma Nursing Research Center, Kashan University of Medical Sciences, Kashan, IR Iran

**Keywords:** Chronic disease, Nursing care

Many people live their lives with chronic conditions. Eighty percent of people who seek medical treatments suffer from chronic diseases (1). In USA 26% of adults have more than one chronic condition such as arthritis, hypertension, and diabetes (2). Many times patients have persistent or recurrent pain (chronic non-cancer pain) which make the condition worse. Chronic non-cancer pain causes significant morbidity, interfering with a patient's ability to perform activities of daily living, family life, and employment, and is in associate with significant psychological stress (3).Chronic conditions effect patients, their families, communities and health systems. Sometimes patients and their families are facing continual and perdurable struggle to control the disease or its complications (4). Frustration might be a result of these continuous and endless struggles. Heart failure is also amongst one of the most disturbing chronic conditions with different consequences. In this issue Ghanbari et al. have presented result of a study on cognitive functions of heart failure patients. These patients are in the risk of frustration (5).

Frustration is a complex emotion and its effects are huge. Sources of frustration include interference with everyday activities, the interruption of life goals and roles and the unpredictability of pain (6). Frustration can have many negative consequences. Sometimes patients stop their self-care activities or even stop their essential prescription. Many times frustration ends up to depression and a deep feeling of anger. People may lose their interest in life. I have heard many times from patients that they prefer to die then continuing their current situation. Patients need more support during these hard times. Nurses can be a source of support for these patients.

How can we help these patients? First, patients should feel our supportive presence (7). We should identify the frustration and its causes. Patients should feel that we are beside them not in front of them. We should never blame patients for their non-compliance behaviors but we should understand their reactions and see what we can do.

Second, patient should feel that they are in good hands and under best possible treatments. Maybe we should go to details of patients' management. The physicians should be available easily. The difficulty of getting prescription medications and lack of continuity of care make the patients more frustrated (8).

Third, the peer supported groups can be a great help, especially for children. They can share their feelings and experiences and support each other (9). Support groups are groups of people who gather to share common problems and experiences associated with a particular problem, condition, illness, or personal circumstance. In a support group, people are able to talk with other folks who are like themselves -people who truly understand what they're going through- and can share the type of practical insights that can only come from a firsthand experience (10). Nurses can be active in creating these supportive groups. 

The last and maybe the most important action is that we should move from disease-focused model of care, to patient-centered models (11). These models have a long history and strong roots in nursing profession but still their implementation in every-day care settings seem to be far away from reality.

Finally frustration is an important concept in chronic care that even can be suggested as a new nursing diagnosis. This condition needs to be identified and professional interventions need to be implemented to support patients in their long and painful struggle.
